# Editorial: Exploring the role of microorganisms in silages: species, communities, interactions, and functional characteristics

**DOI:** 10.3389/fmicb.2023.1196267

**Published:** 2023-05-30

**Authors:** Siran Wang, Qing Zhang, Lin Sun, Ashwani Kumar

**Affiliations:** ^1^Institute of Ensiling and Processing of Grass, College of Agro-Grassland Science, Nanjing Agricultural University, Nanjing, China; ^2^College of Forestry and Landscape Architecture, South China Agricultural University, Guangzhou, China; ^3^Inner Mongolia Engineering Research Center of Development and Utilization of Microbial Resources in Silage, Inner Mongolia Academy of Agriculture and Animal Husbandry Science, Hohhot, China; ^4^Metagenomics and Secretomics Research Laboratory, Department of Botany, University of Allahabad (A Central University), Prayagraj, Uttar Pradesh, India

**Keywords:** silage, fermentation products, bacterial community, functional characteristics, ensiling

Ensiling of herbages is popular throughout the world to supply quality feeds for ruminants. This process involves creating an anaerobic environment for lactic acid bacteria (LAB) to convert fermentable substrates to abundant organic acids, mainly lactic acid, resulting in an acidic environment in silo. During ensiling, the accumulation of lactic acid is mainly responsible for decreased pH (< 4.2), ensuring good conservation of herbages for a long period. Numerous studies have investigated the relationships between fermentation products and microbial community structure during ensiling through Next Generation Sequencing technology. Whereas, most of the studies only describe the microbial compositions and overlook their functionality during ensiling. To our knowledge, the microbial function usually plays a more critical role than their compositions in regulating silage fermentation products. Therefore, an investigation should be conducted to study the microbial role in silage. This topic explores the species, community compositions, interactions, and functional characteristics of microbial communities in silages. Our Research Topic comprises 34 original research articles contributed by more than 200 authors covering the application of different harvest frequencies on microbial community structure and metabolic properties of annual ryegrass silage (Fu et al.); in this Research Topic, Fu et al. assessed the impacts of various harvest frequencies on fermentative products, microbial community compositions and function, and their metabolites in ryegrass. They combined the single-molecule real-time (SMRT) sequencing method with the ultra-high-performance liquid chromatography-mass spectrometry (UHPLC-MS/MS) technique to analyze the bacterial community and metabolomic characteristics.

Paper mulberry (*Broussonetia papyrifera* L.), a typical and widely distributed woody forage, is fast growing; rich in crude protein [211~246 g/kg dry matter (DM)], amino acids and flavonoids; and widely distributed in Asia. Cheng et al. isolated and identified LAB strains from different resources, and assessed their influences on the fermentation products, nutritive value, and microbial community structure of paper mulberry. Three of these LAB strains (*Lactiplantibacillus plantarum*, YC1; *Levilactobacillus brevis*, PC3; and *Lactiplantibacillus plantarum*, BP17) and one commercial inoculant were used, and found that PC3 and BP17 can improve the fermentation quality of paper mulberry silage and could be used as silage starter cultures.

Chen et al. investigated the improvement in the quality of Napier grass silage with pyroligneous acid. Pyroligneous acid (PA), a by-product of biochar production, is known to have strong antimicrobial and antioxidant activities. In this investigation, PA treatment reduced the levels of yeasts, molds, and coliform bacteria as well as the pH and ammonia nitrogen (NH_3_-N) content. The addition of PA decreased the relative abundance of *Klebsiella* and *Kosakonia* while increasing *Lactobacillus*. PA application could improve fermentation characteristics and aerobic stability, as well as alter the microbial communities of silage. According to a study by Du et al., LAB might directly alter the chemical contents and fermentation quality of native grass silage by modifying the bacterial community. To achieve high-quality silage, the complex LAB (*Lactobacillus plantarum, Lactobacillus buchneri*, and *Pediococcus pentosaceus*) showed the potential ability to reduce pH and increase the relative abundance of LAB through synergistic actions. These findings offered a theoretical foundation for the deployment of inoculants in native grass and suggested that the complex LAB might enhance the ensiling performance of native grass silage.

Sorghum grows as one of the top five cereal crops with an annual global planting area and yield. As the sorghum plant is low in the ratio of the crop to stalk (0.5–0.8), sorghum production would likely accompany by a large amount of sorghum stalk, which could be an important feedstock for industrial production or feedstuff for livestock husbandry if properly used. Indeed, it is used as a critical roughage for ruminants feeding, acting as an important energy source via microbial fermentation in the rumen. Zhang et al. described the fermentation products, chemical and bacterial compositions in sorghum stalk silages. They concluded that the succession of fermentation parameters, nutrient components and bacterial community indicated a successful dominant establishment of LAB and a fast advent of fermentation plateau, and the high-moisture sorghum stalk could be ensiled directly, but the pH of mature silage is a little high.

Sweet sorghum is an important crop in arid and semi-arid climatic regions, which can tolerate adverse environments such as limited rainfall, high temperature, and low soil fertility. Xu et al. studied the effects of LAB additives on fermentative parameters and loss, and bacterial compositions in sweet sorghum silages at various silo densities. They found that sweet sorghum silage showed satisfactory fermentation quality, with a density of no < 650 kg/m^3^, and inoculating LAB improved fermentation quality and reduced fermentation weight loss. *Lactiplantibacillus* and *Lentilactobacillus* presented as minor taxa in fresh sweet sorghum and dominated the bacterial community of all silages; inoculating LAB and increasing silo density can contribute to the decreasing *Lactiplantibacillus* abundance and increasing *Lentilactobacillus* abundance.

Ensiling is a complex biochemistry process determined by several factors, including temperature, moisture, raw materials nutritional compositions, harvest time, raw materials length, pack density, the microbiome in raw materials, and others. In particular, preserving silage from forages and grasses depends on the microbial ecological diversity and the epiphytic LAB play a determining role in high-quality silage conservation. Du et al. studied the bioaugmentation impacts of some LAB additives on fermentation characteristics and bacterial community structure and functions in native grass silage. They found that the complex LAB (*Lactobacillus plantarum, Lactobacillus buchneri*, and *Pediococcus pentosaceus*) could improve the ensiling performance of native grass silage, and lay a theoretical basis for inoculant application in native grass.

Alfalfa is a high-yielding and nutrient-rich forage legume and is widely used as a dietary component of ruminants. Using alfalfa in ruminant production can expand the protein source and thus reduce the dependency on expensive protein supplements. Bao et al. studied the impacts of bioaugmented silage with *Pediococcus pentosaceus* and laccase on ensiling performance, chemical compositions, enzymatic hydrolysis, and bacterial communities in alfalfa silage. They concluded that the bioaugmented ensiling with laccase and *Pediococcus pentosaceus* combination could be an effective and practical strategy to improve silage fermentation and nutrient preservation of alfalfa silage.

Purple perilla (*Perilla frutescens* L.), an annual short-day medicine food homology plant that belongs to the family *Lamiaceae* and the genus *Perilla*, is commonly available in many countries and has been cultivated in China for more than 2,000 years. With a large variety of functional ingredients such as essential oil, polyphenol, flavone and so on, purple perilla shows antimicrobial, antioxidants, and healthy functions. Li et al. assessed the impacts of *Perilla frutescens*, alone or combined with citric acid or *Lactobacillus plantarum*, on fermentation products, and fungal and bacterial compositions of forage oat during ensiling and air exposure. They found that *Perilla frutescens*, alone or in combination with citric acid, can improve the aerobic stability of forage oat silage by shifting bacterial and fungal community composition, and can be used as a new additive to prepare high-quality silage for animal production.

Bunker silos, round bales and silage bags are becoming common ways to store silage. However, a lack of scientific ensiling management usually results in poor chemical composition and excessive butyric acid contents. Xia et al. evaluated the impacts of silage bags, round bales, and bunker silos on the chemical compositions, ensiling characteristics, mycotoxin contents, aerobic stability, and microbial community structure of whole-crop maize silages. Considering the feed value and food safety of silage in the feeding process, silage bags are recommended for whole-crop maize silages according to the observed nutritional quality, fermentation index and mycotoxin content.

Alongside hay, native grass is regarded as an important resource for animal feed in the Mongolian Plateau, and a large quantity is produced annually in pastoral areas. Hou et al. characterized and identified the isolated LAB strains, and studied their effects on ensiling performance of native grass on the Inner Mongolian Plateau. This study revealed that native grass has abundant LAB species and can be well preserved through silage.

With the rapid development of ruminant husbandry, alfalfa (*Medicago sativa* L.) becomes an essential roughage for dairy ration. Ensiling is one of the effective methods to preserve alfalfa nutrients. Most true protein fractions in alfalfa are degraded into non-protein nitrogen during ensiling. In particular, the activity of harmful microorganisms, such as clostridia and enterobacteria, extensively reduces the amino acid content, leading to nutrient losses. Huo et al. investigated the influences of LAB inoculants on fermentation profile, carbohydrate and protein compositions, and bacterial communities in alfalfa silages. They concluded that fermentation quality and nutrient preservation of alfalfa silage were efficiently improved by inoculating with *L. pentosus*.

In summary, this Research Topic provided diverse knowledge on the microbial community in silage from species, community, interaction, and function aspects using multidisciplinary approaches combining multi-omics techniques. This Research Topic of manuscripts provided innovative results, and proved numerous unresolved problems that need further exploration in silage. Nevertheless, there is still a big study gap understanding the effect of microbes on fermentative products and animal response. Hence, future studies should focus on inhibiting undesirable products in silages and animal performance.

## Author contributions

SW and AK wrote and revised the manuscript. All authors have read and approved the final manuscript.

